# Slowly Progressive Rhabdomyolysis Post COVID-19: Insights for Acute Kidney Injury Prediction With Discordant Creatine Kinase and Myoglobin Elevations

**DOI:** 10.7759/cureus.68145

**Published:** 2024-08-29

**Authors:** Takeshi Okubo, Hidemichi Kouzu, Ayaka Kamada, Kota Endo, Wataru Kawaharata, Wataru Ohwada, Kentaro Suda, Nobutaka Nagano, Ayami Sakurai, Masayuki Koyama, Masato Furuhashi

**Affiliations:** 1 Department of Cardiovascular, Renal and Metabolic Medicine, Sapporo Medical University School of Medicine, Sapporo, JPN; 2 Department of Clinical Genetics, Sapporo Medical University Hospital, Sapporo, JPN

**Keywords:** creatine kinase, myoglobin, rhabdomyolysis, covid-19, sars-cov-2

## Abstract

Rhabdomyolysis can lead to acute kidney injury (AKI), primarily due to myoglobin-induced tubular damage. We present a case of slowly progressive rhabdomyolysis following SARS-CoV-2 infection in a 28-year-old male who was monitored through serial serum creatine kinase (CK) and myoglobin levels. Despite prominent CK elevations, the patient did not develop AKI, probably due to disproportionately mild serum myoglobin elevation with distinctive cyclic spikes. This case underscores the informative value of frequent monitoring of both CK and myoglobin to assess muscle damage severity and AKI risk in rhabdomyolysis, particularly with viral infections like COVID-19 that can cause delayed-onset muscle injury.

## Introduction

Rhabdomyolysis, caused by acute skeletal muscle damage by various triggers such as trauma, exertion, drugs, toxins, and infections, leads to elevated levels of serum creatine kinase (CK), predominantly its skeletal muscle isoenzyme, CK-MM, and myoglobin, both of which localize in the cytoplasm of striated muscle where they play crucial roles in energy metabolism [1]. One of the serious complications of rhabdomyolysis is acute kidney injury (AKI), caused primarily by myoglobin excreted through glomerular filtration, which induces tubule obstruction and oxidant injury [2]. Serum CK and myoglobin levels, mostly reflecting the initial muscle damage and usually normalizing within a few days once the injury ceases, have been shown to predict subsequent renal dysfunction [3-5]. However, it remains unclear how patients with rhabdomyolysis should be monitored and managed when muscle injury persists during the acute phase.

Here, we present a case of slowly progressive rhabdomyolysis after SARS-CoV-2 infection in which serial measurements of serum CK and myoglobin levels were conducted. Our observations provide insights for assessing the risk for the development of AKI in rhabdomyolysis with an unusual clinical course.

## Case presentation

A 28-year-old male was transported to the emergency room due to fatigue. He had a history of craniopharyngioma surgery 10 years ago and had been receiving hormone replacement therapy for panhypopituitarism, including hydrocortisone at 10 mg quaque die (QD), levothyroxine sodium at 75 µg QD, desmopressin at 120 µg QD, and somapacitan at 1.5 mg weekly. He was also receiving ezetimibe and febuxostat. One day before admission, he was diagnosed with COVID-19. Because of repeated vomiting and fatigue, he was unable to take his oral medications for two days. 

**Figure 1:**
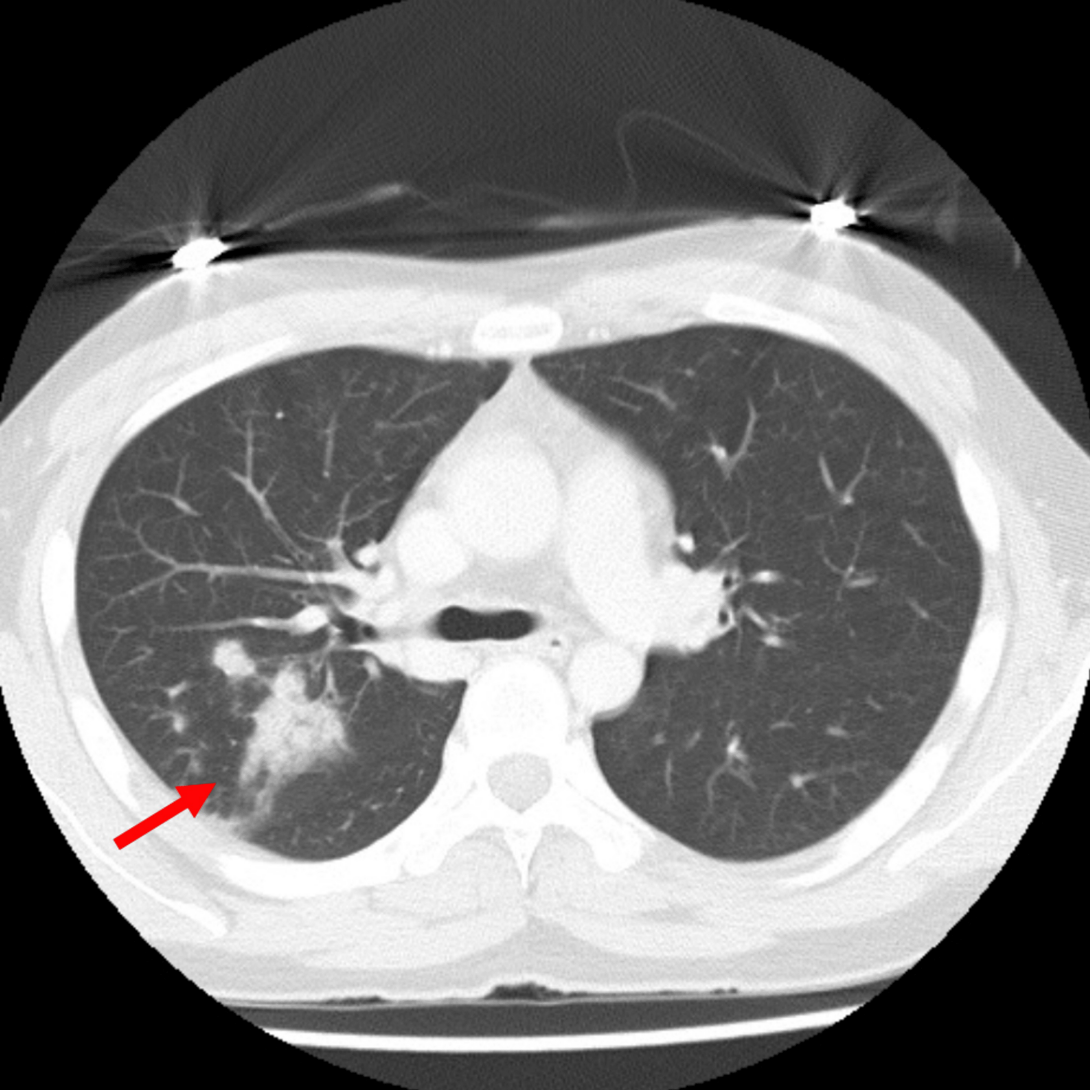
Chest CT on admission showing infiltrates in the right lung (red arrow)

Upon arrival, he presented with shock vitals: body temperature of 37.5°C, heart rate of 133 beats per minute, blood pressure of 75/47 mmHg, and 80% oxygen saturation on room air. A physical examination showed no remarkable findings except for overweight (body mass index of 26.4). A chest CT scan showed infiltrates in the right lung, suggesting bacterial pneumonia (Figure 1). Initial blood tests revealed elevated C-reactive protein (CRP) and procalcitonin levels, liver dysfunction, and severe renal impairment likely due to dehydration from central diabetes insipidus (Table 1). The free thyroxine level was within the normal range. A low cortisol level and unstable hemodynamics strongly indicated acute adrenal insufficiency triggered by COVID-19. Eosinopenia under the condition of adrenal insufficiency was likely to be explained by acute infection and shock-mediated catecholamine secretion [6]. The patient was admitted to the intensive care unit (ICU). We started treatments with aggressive fluid administration, intravenous hydrocortisone (100 mg every eight hours for four days, followed by stepwise tapering) for acute adrenal insufficiency, sulbactam/ampicillin (3 g twice daily) for potential bacterial co-infection, and remdesivir (200 mg on the first day, followed by 100 mg daily) as a specific antiviral treatment for COVID-19. After the initial administration of hydrocortisone, his cortisol level was sufficiently elevated (31.00 μg/dL). During acute treatments, hypernatremia (~156 mmol/L) and hypokalemia (~3.0 mmol/L) were occasionally observed, and they were tightly corrected. The patient's condition stabilized quickly, and the CRP level decreased steadily.

**Table 1:** Laboratory data ACTH: Adrenocorticotropic hormone, ALP: Alkaline phosphatase, ALT: Alanine aminotransferase, AST: Aspartate aminotransferase, BUN: Blood urea nitrogen, Ca: Calcium, CH50, CK: Creatine kinase, Cl: Chlorine, Cre: Creatinine, CRP: C-reactive protein, D-BIL: Direct bilirubin, FT3: Free triiodothyronine, FT4: Free thyroxine, γ-GTP: Gamma-glutamyl transferase, K: Potassium, LDH: Lactate dehydrogenase, Na: Sodium, NAG: N-acetyl-β-D-glucosaminidase, P: Inorganic phosphate, T-BIL: Total bilirubin, TSH: Thyroid-stimulating hormone

Parameters	Results	Reference range
Blood count / Biochemistry / Endocrine / Coagulation (On admission)		
White blood cell	11,300	3,300-8,600 /μL
Neutrophil	63.0	36.0-69.8%
Lymphocyte	29.8	21.0-52.2%
Monocyte	6.9	4.5-10.6%
Eosinophil	0.0	0.8-10.1%
Basophil	0.3	0.0-1.1%
Red blood cell	435	435-555 ×10⁴ /µL
Hemoglobin	13.8	13.7-16.8 g/dL
Platelet count	10.4	15.8-34.8 ×10⁴ /µL
Total protein	7.9	6.6-8.1 g/dL
Albumin	4.0	4.1-5.1 g/dL
T-BIL	1.9	0.4-1.5 mg/dL
D-BIL	0.7	0.0-0.2 mg/dL
AST	320	13-30 U/L
ALT	411	10-42 U/L
LDH	460	124-222 U/L
ALP	378	110-370 U/L
γGTP	241	13-64 U/L
Glucose	130	73-109 mg/dL
CK	749	59-248 U/L
CK-MB	7.3	0.0-4.9 ng/mL
Myoglobin	651	28-72 ng/mL
Uric acid	11.8	3.7-7.0 mg/dL
BUN	22	8-20 mg/dL
Cre	3.08	0.65-1.07 mg/dL
Na	144	138-145 mmol/L
Cl	109	101-108 mmol/L
K	3.1	3.6-4.8 mmol/L
Ca	9.0	8.8-10.1 mg/dL
P	4.0	2.7-4.6 mg/dL
CRP	12.63	0.00-0.14 mg/dL
Procalcitonin	75.3	<0.5 ng/mL
ACTH	11.7	7.2-63.3 pg/mL
Cortisol	1.42	7.07-19.60 μg/dL
TSH	0.04	0.50-5.00 μIU/mL
FT3	2.06	2.30-4.00 pg/mL
FT4	1.11	0.90-1.70 ng/mL
D-dimer	2.5	<1.0 μg/mL
Urinary β2-microglobulin	4,839	<289 μg/L
Urinary NAG	31.6	<11.5 U/L
Immunology (Day 7)		
IgG	1109	861-1747 mg/dL
IgA	262	93-393 mg/dL
IgM	29	33-183 mg/dL
CH50	50.8	31.6-57.6 U/mL
C3	98	73-138 mg/dL
C4	20	11-31 mg/dL
Anti-nuclear antibody titer	<1:40	<1:40
Anti-MDA5 antibody	<4	<32 index
Antii-Mi-2 antibody	<4	<53 index
Atti-TIF1-γ antibody	<5	<32 index
Anti-ARS antibody	<5	<25 index

**Figure 2:**
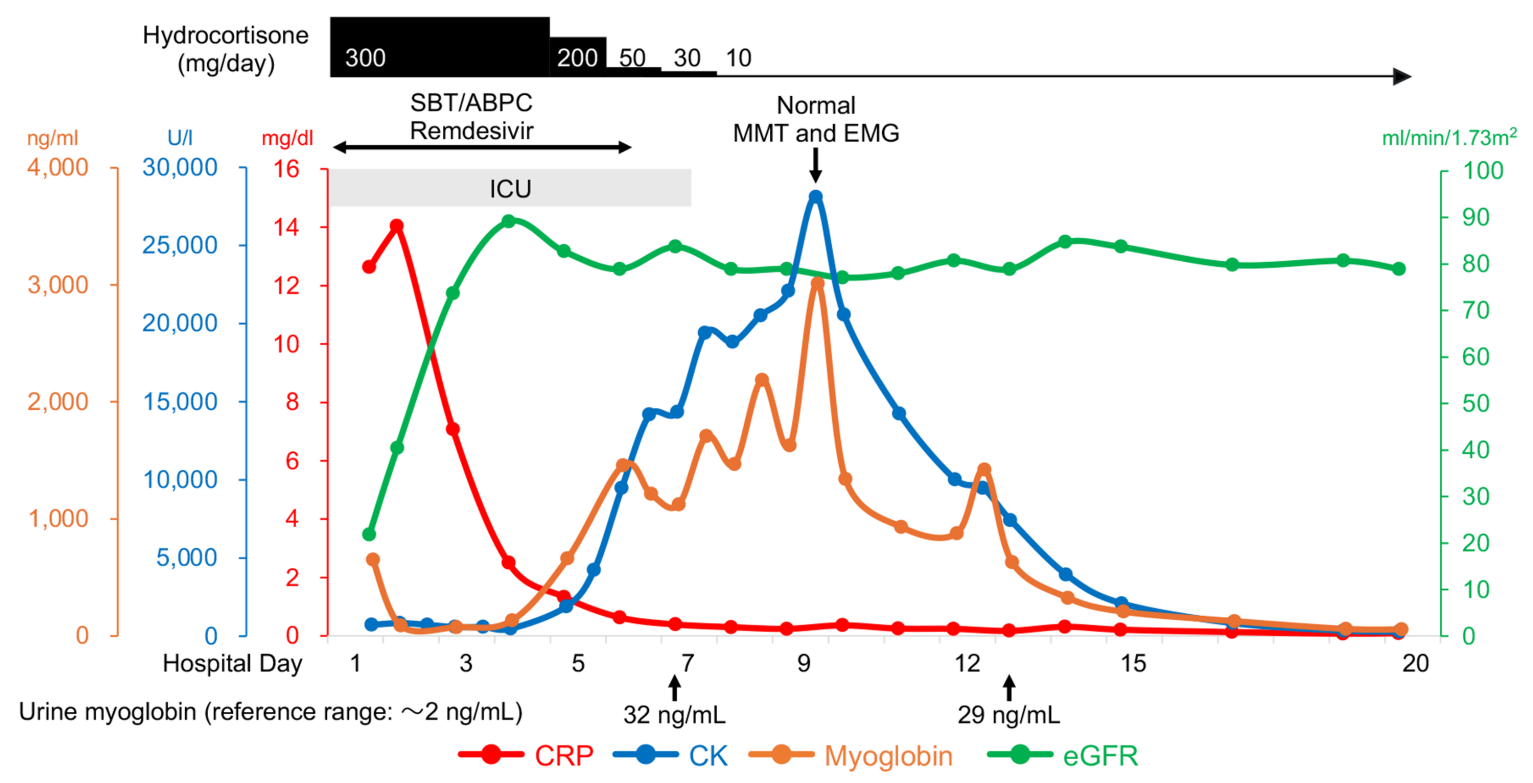
Clinical course of the patient after admission CK: Creatine kinase, CRP: C-reactive protein, eGFR: Estimated glomerular filtration rate, EMG: Electromyography, MMT: Manual muscle testing, SBT/ABPC: Sulbactam/ampicillin

**Figure 3:**
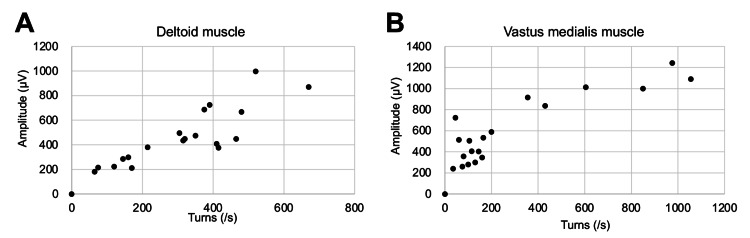
Needle electromyography results on the day of peak creatine kinase level Normal linear relationships between frequency and amplitude in the right deltoid muscle (A) and the left vastus medialis muscle (B) are observed.

Since the initial blood examination showed renal dysfunction with mild CK and myoglobin elevation and a urinary test showed elevated β2-microglobulin and N-acetyl-β-D-glucosaminidase, markers of renal tubular damage (Table 1), we monitored CK and myoglobin levels in daily routine blood examinations during the ICU stay. Despite overall clinical improvement, the patient's serum CK level gradually increased. We stopped sulbactam/ampicillin and remdesivir on the sixth day, but the CK level continued to rise and peaked at 28,140 U/L on the ninth day (Figure 2). Isoenzyme examination revealed that CK-MM accounted for 100% of the total CK activity, indicating that the elevated CK level was derived exclusively from skeletal muscle. Immunology tests for autoimmune myositis were all negative (Table 1). Notably, this prominent CK elevation occurred without any symptoms or decrease in muscle strength, as indicated by a full score in manual muscle testing. Additionally, needle electromyography of the right deltoid and left vastus medialis muscles revealed no spontaneous activity at rest and normal recruitment and interference patterns without abnormalities in amplitude or frequency under voluntary contraction, thus providing no electrophysiological evidence suggestive of inflammatory myopathy (Table 2, Figure 3). We simultaneously monitored serum myoglobin with CK twice daily (06:00 a.m. and 12:00 p.m.) and noticed periodic spikes of myoglobin, indicating recurrent muscle injuries during short periods. His physical activity was limited by sequestration due to COVID-19, ruling out muscle overuse. Curiously, serum myoglobin levels increased to only about 3,000 ng/mL, disproportionately lower than the CK levels. Qualitative urinary dipstick tests were repeatedly negative for heme detection, and urine myoglobin was only slightly elevated (~32 ng/mL). Following the restoration of the initial dehydration, renal function remained stable, and the patient was discharged on the 20th day of hospitalization. The level of CK on discharge (323 U/L) was still above the upper normal threshold, but it was normalized on a follow-up visit two weeks later and has been within the normal range for one year.

**Table 2:** Measurement values of frequency and amplitude of electromyography with gradual increases in the intensity of voluntary contraction

Deltoid muscle		Vastus medialis muscle	
Turns (/s)	Amplitude (μV)	Turns (/s)	Amplitude (μV)
0	0	0	0
65	181	35	241
75	215	45	724
120	224	60	514
145	285	75	261
160	298	80	356
170	211	100	281
215	380	105	505
305	496	115	407
315	436	130	299
320	449	145	404
350	475	160	347
375	687	165	534
390	725	200	590
410	408	355	917
415	377	430	837
465	449	605	1014
480	668	850	1000
520	997	975	1243
670	870	1055	1092

## Discussion

Rhabdomyolysis is commonly induced by trauma, strenuous exercise, alcohol abuse, prolonged immobilization, and lipid-lowering drugs such as statins and fibrates [2], which were not confirmed in this case. Moreover, there is no strong evidence in the literature linking remdesivir or sulbactam/ampicillin, which were used for the acute phase treatments in this case, to rhabdomyolysis. Adrenal insufficiency complicating rhabdomyolysis is extremely rare, and it was reported to have resolved rapidly with steroid therapy in one case [7]. Severe hypothyroidism can also occasionally induce rhabdomyolysis, but most cases are due to untreated chronic thyroiditis [8]. Thus, deterioration of panhypopituitarism is also unlikely to have been the primary cause of rhabdomyolysis in our case.

Viral infections, particularly influenza virus infections, have been reported to induce rhabdomyolysis [2], though the mechanisms have yet to be determined [9]. Emerging evidence has also demonstrated that COVID-19 causes rhabdomyolysis. The prevalence of rhabdomyolysis in hospitalized patients with COVID-19 has been reported to be from 0.2% to 2.2% and 4.0% [10,11]. The mechanisms by which COVID-19 provokes rhabdomyolysis are assumed to include local inflammation induced by direct virus invasion through the angiotensin-converting enzyme 2 (ACE2) expressed on skeletal muscle and disruption of muscle homeostasis secondary to systemic changes in cytokines, including interleukin-6 [10]. Since our patient was diagnosed as having COVID-19 one day before admission and no other causes were confirmed, COVID-19 is likely to have been the primary trigger of rhabdomyolysis.

A previous literature review covering 86 cases of COVID-19-associated rhabdomyolysis revealed that patients were predominantly male (77%) and the average age of the patients was 50 years, with the median peak CK being 15,783 U/L [10]. About 50% of the patients were complicated with hypertension, diabetes mellitus, or obesity, but younger patients like ours, as well as children, also developed rhabdomyolysis. In a single-center retrospective study, 140 consecutive hospitalized patients with COVID-19 who had CK >1,000 U/L during admission were analyzed [12]. In that cohort, 94% of the patients had a peak CK on presentation or within one week of admission, suggesting that muscle injury mostly develops early after infection. In contrast, our patient slowly developed rhabdomyolysis after admission, and the CK level reached a peak on the ninth day and remained above the normal threshold throughout the hospital admission. Late development of rhabdomyolysis after COVID-19, as in our case, has been occasionally reported [13]. Besides COVID-19, the pandemic of novel swine-origin influenza H1N1 in 2009 was associated with a high prevalence of elevated CK [14], occasionally leading to severe rhabdomyolysis [15]. Physicians should be aware that severe rhabdomyolysis can occur during viral infections, especially during emerging virus outbreaks, even as a late complication.

The mechanisms underlying the varying onset timing of rhabdomyolysis in different COVID-19 cases remain unclear. We did not perform a muscle biopsy due to normal electromyography findings. However, a recent histological study of autopsy cases has suggested that COVID-19 may induce immune-mediated myopathy rather than direct viral invasion of muscle tissue [16]. This evidence raises the possibility that the corticosteroids administered for acute adrenal insufficiency in our patient might have suppressed the initial skeletal muscle inflammation, leading to delayed muscle enzyme leakage.

The prevalence of AKI in COVID-19-associated rhabdomyolysis has been reported to be high, ranging from 52% to 69% [10,11]. Although elevation of the CK level above 10,000 U/L, the threshold for severe rhabdomyolysis [17], unexpectedly persisted for seven days, AKI did not develop in our patient, with only a marginal increase in serum creatinine from 0.85 mg/dL (day 4) to 0.97 mg/dL (day 10) after the restoration of initial dehydration. Additionally, our patient remained completely asymptomatic and had no muscle weakness. It has been shown that the correlation between CK level and incidence of AKI is generally weak, and the predictive role of CK level for renal replacement therapy or in-hospital mortality is limited [2,3,17].

Myoglobin is the direct pathogenic molecule causing AKI during rhabdomyolysis [2]. However, it is often not monitored or even measured due to its short half-life (two to three hours) compared to that of CK (1.5 days). The early phase of rhabdomyolysis can be detected by measurement of the level of myoglobin, but the level normalizes rapidly once muscle injury ceases [1]. In our case, we monitored not only CK but also serum myoglobin twice daily, allowing us to detect the concealed recurrent muscle injury. Notably, despite a prominent increase in CK level, the elevation of serum myoglobin level was disproportionately mild and remained below the reported threshold (4,000 to 8,000 ng/mL) for AKI risk [4,5]. In severe rhabdomyolysis, in which AKI develops in approximately 50% of cases, cases with a myoglobin/CK ratio exceeding 0.2 were reported to be at risk for AKI [18]. Several possibilities may explain the low myoglobin/CK ratio of 0.11 in our case: 1) accumulation of CK due to its long half-life may have caused an overestimation of ongoing muscle damage; 2) activation of extrarenal myoglobin metabolism, possibly in the liver and spleen, may have led to only a mild increase in serum myoglobin concentrations [4,19]; and 3) CK, which has higher permeability of the myocyte membrane than myoglobin, may have leaked more predominantly, as suggested in acute psychotic patients with rhabdomyolysis [20]. These factors may have contributed to the low renal excretion of myoglobin and the absence of AKI. However, the scarcity of studies simultaneously evaluating both markers limits our understanding of the precise mechanisms underlying this CK and myoglobin dissociation. Future prospective studies are needed to elucidate scenarios where such dissociation occurs as well as its predictive role for AKI development.

## Conclusions

Delayed-onset rhabdomyolysis can occur as a late complication of viral infections, including COVID-19, emphasizing the need for extended clinical monitoring. Our case demonstrates the potential value of dual monitoring of myoglobin and CK in assessing muscle damage severity and AKI risk in persistent rhabdomyolysis. Our findings suggest that disproportionately mild myoglobin elevations, even with prominent CK elevations, may indicate a lower AKI risk. The observed cyclic myoglobin spikes highlight the dynamic nature of muscle injury following viral infections. While our observations suggest that the myoglobin/CK ratio could be useful as a risk assessment tool, further studies are needed to determine the scenarios that would benefit from the dual monitoring for predicting prognosis and guiding treatment decisions in rhabdomyolysis, as well as studies to clarify the mechanisms underlying prominent CK elevation without apparent skeletal muscle clinical manifestations.
